# Common Biological Mechanisms of Alcohol Use Disorder and Post-Traumatic Stress Disorder

**DOI:** 10.35946/arcr.v39.2.04

**Published:** 2018

**Authors:** Junghyup Suh, Kerry J. Ressler

**Affiliations:** Junghyup Suh, Ph.D., is an instructor in the Department of Psychiatry, Harvard Medical School, Boston, Massachusetts, and an assistant neuroscientist in the Division of Depression and Anxiety Disorders, McLean Hospital, Belmont, Massachusetts. Kerry J. Ressler, M.D., Ph.D., is a professor in the Department of Psychiatry, Harvard Medical School, Boston, Massachusetts, and the chief scientific officer and chief of the Division of Depression and Anxiety Disorders, McLean Hospital, Belmont, Massachusetts

**Keywords:** addiction, animal models, depression, neural circuitry, post-traumatic stress disorder (PTSD), stress, trauma

## Abstract

Post-traumatic stress disorder (PTSD) and alcohol use disorder (AUD) are highly comorbid. Although recent clinical studies provide some understanding of biological and subsequent behavioral changes that define each of these disorders, the neurobiological basis of interactions between PTSD and AUD has not been well-understood. In this review, we summarize the relevant animal models that parallel the human conditions, as well as the clinical findings in these disorders, to delineate key gaps in our knowledge and to provide potential clinical strategies for alleviating the comorbid conditions.

Alcohol use disorder (AUD) is one of the most common co-occurring disorders among individuals diagnosed with post-traumatic stress disorder (PTSD).^1^ Many people who have PTSD use alcohol in an attempt to ameliorate debilitating symptoms such as anxiety and hyperarousal. Clinical and epidemiological studies have consistently reported that PTSD is associated with a threefold higher risk for developing AUD, and for individuals who have PTSD, the lifetime prevalence of AUD has been estimated at 40%.^2^ The severity of PTSD symptoms is positively related to the level of alcohol use, and it also predicts alcohol craving in response to trauma- and alcohol-related cues. Despite the high rates of comorbidity, there is a substantial gap in understanding how traumatic experience leads to transition from initially controlled alcohol consumption (reward phase) to the development of alcohol-seeking and dependence (negative reinforcement phase). This review summarizes clinical observations and highlights findings from preclinical animal models, and focuses particularly on the alterations and dysfunctions in neural circuitry and stress hormone systems that may underlie enhanced vulnerability to AUD in context of PTSD (Figure 1).

## Preclinical Models of PTSD and AUD

### Animal model approaches

There are several procedures commonly used to create animal models of stress or PTSD and to employ stress components that are known to lead to enhanced risk for AUD.^3^ Many procedures are simple, easy to implement, and effective at inducing a broad departure from endocrinological, physiological, and neurobiological homeostasis.^4^ Also, both acute and chronic stressors can lead to physical and psychiatric pathology. First, we briefly describe a range of stress-related approaches to modeling the phenotypes of PTSD and AUD. Then, we review supporting studies in more detail, examining common biological components of both disorders.

Widely used physical stressors include exposure to immobilization, restraint, cold-water swimming, electric footshocks, and noxious stimuli.^4^ Immobilization or restraint stress commonly is produced by confining a naïve animal inside a bag or tube. Also, relevant naturalistic or ethological stressors have been used to trigger stress states.^4^ Models of psychological stress include exposure to predator odor; an elevated platform; or a bright, open area; whereas models of social stress include social isolation, maternal deprivation, and social defeat. In some studies, more than one stressor is applied concomitantly to test the generality of a hypothesized mechanism or to enhance the intensity of desired responses.

Alcohol behaviors include various responses and changes elicited by alcohol exposure and withdrawal. Examples of these behaviors are alcohol craving, compulsive alcohol-seeking, excessive alcohol intake, alcohol dependence, and relapse. In this review, we survey the recent progress in animal modeling for two main aspects of AUD-related alcohol behaviors—alcohol consumption and alcohol-seeking. In general, experiments designed to investigate the effects of stress and alcohol behaviors can be divided into three categories. In the first category, alcohol-naïve animals experience stress, then alcohol is introduced concurrently or after an incubation period.^5^–^7^ In the second category, animals are familiarized to alcohol or to drinking alcohol before stress is introduced.^8^ In the third category, animals develop alcohol behaviors, subsequently extinguish those behaviors, and then stress is introduced during a development, extinction, or reinstatement period.^9^ In these experimental designs, alcohol behaviors are generally monitored through preference ratios and by measuring intake. Typically, animals have free access to water or an alcohol solution, and alcohol preference and intake are determined by the amount of liquid consumed and the number of approaches.

A considerable body of evidence suggests that stress triggers negative affective states and subsequent adaptive changes that lead to the development of AUD, so many animal models for AUD have focused on creating a condition in which a stress procedure precedes alcohol exposure (or re-exposure).^3^ Notably, however, it also has been suggested that excessive drinking is a risk factor for developing anxiety disorders such as PTSD. There are several reasons this may be the case. One possibility is that in cortical regulatory areas such as the medial prefrontal cortex (mPFC), impairments from excessive drinking are similar to impairments from repeated stress. For example, in a 2012 study of mice, Holmes and colleagues examined the effects of chronic alcohol exposure on the prefrontal cortex (PFC) and its capacity to mediate fear extinction.^8^ Fear extinction is a reduction in the frequency or intensity of a conditioned fear response (e.g., freezing) after repeated presentation of a conditioned stimulus (e.g., a sound) in the absence of the unconditioned aversive stimulus (e.g., a footshock). Holmes and colleagues found that mice intermittently exposed to continuous vaporized alcohol had significant remodeling of mPFC neurons and demonstrated impaired fear extinction.^8^

Using a combination of these preclinical models and molecular, genetic, and pharmacologic manipulation approaches, recent investigations have made great strides in delineating the neurobiological processes underlying stress-induced escalated alcohol intake or alcohol-seeking behavior. Next, we summarize some details of these models and their relevance to both disorders, as well as to comorbid PTSD and AUD.

### Restraint or immobilization stress

Restraining rodents in small tubes or on a platform in an acute or chronic manner leads to increased manifestations of anxiety and changes in neuronal morphology within brain regions that mediate fear and anxiety.^10^,^11^ In previous studies, acute immobilization stress in mice significantly elevated hypothalamic pituitary adrenal (HPA) axis activity, resulting in impaired fear extinction and extinction retention following Pavlovian fear conditioning.^12^,^13^ Furthermore, exposure to this stressor led to impaired long-term declarative memory and enhanced anxietylike behavior.^14^

Because of the practical simplicity of restraint-related procedures, numerous studies have employed them to elucidate the relationship between stress and alcohol consumption. However, the results are not conclusive. In some cases the stressor significantly increased alcohol intake, whereas in others alcohol consumption decreased or did not change.^15^,^16^ Therefore, although researchers have speculated about many factors, such as time, individual differences, and stress-induced long-term sensitization or desensitization of the HPA axis,^17^ there appears to be no clear primary determinant on the outcome in those studies.

### Social stress

Social isolation, such as maternal deprivation, is a demonstrated risk factor for alcohol consumption during adolescence and adulthood, particularly in male rats.^18^ In one study, when rat pups were separated from their mothers for 6 hours per day for 20 days, they exhibited increased ethanol consumption during their adolescence, compared with rat pups that had only 15 minutes of deprivation per day. In a similar study, rats (male and female) that experienced a single, 24-hour maternal deprivation on postnatal day 9 and subsequent exposure to restraint stress showed higher ethanol intake than animals that experienced only a single maternal deprivation.^19^ Furthermore, isolation stress during adolescence seemed to similarly increase alcohol consumption. For example, rats housed individually during adolescence exhibited increased ethanol intake and ethanol preference during adulthood.^20^ Moreover, when an intermittent procedure was used to offer these rats alcohol, they drank significantly more ethanol solution and obtained higher blood ethanol levels than rats that received a continuous procedure. In addition, when induced by chronic early life stress, the increase in ethanol consumption lasted for at least 8 weeks.^21^ Notably, the stressed rats displayed a significant deficit in fear extinction but not in fear memory acquisition.

Also, several studies have shown through self-administration and place-conditioning paradigms that exposure to social defeat stress induced escalation of alcohol consumption as well as reinstatement of alcohol-seeking behavior after extinction.^22^ Procedures for invoking social stress can be divided into acute versus repeated, or agonistic encounters in a neutral environment versus resident or intruder settings. In these stress paradigms, the observation of escalated alcohol intake is related to when the stress experience occurred. The animals showed no significant change in alcohol consumption immediately after stress, but they showed an increase 2 hours after stress.^22^

More recent studies with mice demonstrated that a 10-day social defeat stress experience increased ethanol drinking and preference for at least 20 days after the defeat.^6^,^7^ Elevated alcohol consumption was correlated with plasma corticosterone levels and was modulated by the signaling pathway of corticotropin releasing hormone receptor 1 (*CRHR1*) in the ventral tegmental area (VTA) and by dopamine within the nucleus accumbens. Chronic social defeat in rats and mice is wellknown for inducing some core PTSD symptoms, such as increased social avoidance^23^ and anxiety,^22^ as well as enhanced fear memory acquisition.^23^

### Predator-based stress

In rodents, exposure to a natural predator has been shown to provoke high levels of intense fear and stress, followed by long-lasting endocrine and behavioral responses. Typically, the rodents are exposed very briefly (5 to 10 minutes) to a predator or to predator odorants, such as predator urine, which leads to elevation of long-lasting anxietylike behavior.^24^ Specifically, rats exposed to chronic social instability in conjunction with cat odor showed reduced basal glucocorticoid levels, increased glucocorticoid suppression following dexamethasone administration, heightened anxiety, and enhanced fear memory.^25^ These results mimic common endocrine and behavioral measures found in humans with PTSD. Another study demonstrated that rats with higher stress reactivity to predator urine exhibited more alcohol drinking than rats with lower stress reactivity.^5^

### Genetic differences

It has been well-reported that background strain differences can confound stressor reactivity measures and alcohol-related behaviors in the same manner demonstrated for other behavioral measurements, including learning and memory performance, aggression, and emotionality. For example, a phenotypic survey study comparing fear extinction in a panel of inbred mouse strains revealed fear extinction impairment in the 129/SvImJ strain due to a failure in the engagement of corticolimbic extinction circuitry, despite the strain’s normal fear conditioning and nociception.^26^ A similar study showed that chronic exposure to swim stress resulted in a significant decrease in ethanol consumption in mouse strains DBA/2J and BALB/cByJ but not in strain C57Bl/6J, although stress increased sensitivity to the sedative/hypnotic effects of ethanol in all three strains.^27^

## Neurobiological Circuits

Neuroimaging studies have suggested that stress-induced alcohol behaviors may relate to convergent or divergent changes in multiple brain areas. However, to provide a framework for identifying alterations in neural circuitry, we will focus on a few brain areas well-associated with processing fear, anxiety, stress, and rewards. These areas include the amygdala, PFC, hippocampus, and VTA.

### Amygdala

The amygdala is well-known for its role in physiological and behavioral responses to fear, stress, and substance misuse.^5^,^28^,^29^ During fear learning, the amygdala receives multisensory information from the cerebral cortex and thalamus and projects to brain regions that produce behavioral and physiological fear responses.^28^ During fear extinction and fear extinction recall, the mPFC and hippocampus regulate the amygdala from the top down through rich, mutual connections between these areas to modulate previously conditioned fear. Furthermore, severe stress facilitates fear and anxietylike behavior via amygdala-dependent anatomical and physiological changes at synaptic, cellular, and network levels.^4^,^28^,^29^ Neuroimaging studies of healthy humans have shown that increased amygdala activity was evoked by fearful cues and during fear conditioning.^30^ In other studies, combat veterans with PTSD who were exposed to fearful faces exhibited higher levels of amygdala activation than healthy individuals, and they also exhibited hyperreactivity in the presence of trauma-related stimuli.^31^,^32^

In a 2014 study, Garfinkel and colleagues examined amygdala activity in individuals with PTSD.^33^ The researchers used conditioning to generate a fear response to a conditioned stimulus of a colored light (the dangerous context). Later, in a different (safe) context, participants were conditioned to extinguish that fear response. The individuals with PTSD exhibited an increase in amygdala activity when reintroduced to the conditioned stimulus in the safe context, indicating impaired fear extinction. However, in the same study, individuals with PTSD demonstrated low amygdala activity when the extinct conditioned stimulus was reintroduced in the original dangerous context to elicit a fear response (i.e., fear renewal). The low amygdala activity could indicate that these individuals have impaired fear renewal. These findings suggest that individuals with PTSD have a globally diminished capacity to use contextual information to modulate fear expression.

In addition to functional changes, structural changes in the amygdala have been reported in individuals who have PTSD and a history of early life stress. Notably, smaller amygdala and hippocampus volumes have been found in children exposed to different forms of early life stress and have been associated with greater cumulative stress exposure and behavioral problems.^34^ Interestingly, in men who had alcohol dependence, amygdala volume reduction was associated with increased alcohol craving and intake.^35^ Furthermore, it has also been demonstrated that alcohol cues triggered amygdala reactivation in men with alcohol dependence alone,^35^ as well as in individuals who had PTSD and AUD.^31^ However, the neuroimaging data generated by functional magnetic resonance imaging and positron emission tomography do not yet provide the resolution to reliably differentiate amygdala nuclei.

Studies with animal models greatly help extend understanding of the structures and functions of the amygdala in anxiety and fear memory, because the gross anatomy, connectivity, and cellular composition of amygdala nuclei are well-conserved across species.^28^ The amygdala comprises multiple interconnected nuclei that can be classified largely into two groups: cortexlike and striatumlike structures. The cortexlike structure includes the basolateral complex, consisting of the lateral, basolateral, and basomedial amygdala. The striatumlike structure consists of the central nucleus of the amygdala (CeA), which has lateral and medial subdivisions and intercalated cell clusters. During fear conditioning, output activity in the medial division of the CeA is enhanced by excitatory signals originating directly from the lateral amygdala and indirectly through the basolateral amygdala. The output also is modulated by reciprocal connections between the basolateral amygdala and the prelimbic area of the PFC. In contrast, during fear extinction, neural activity in the lateral and basolateral amygdala is reduced, and the infralimbic area of the PFC participates in suppression of fear through the basolateral amygdala and the intercalated cells.

Recent studies suggest functional and molecular heterogeneity for the cell types and projections within some of the amygdala subnuclei. For example, in one of our studies, we found that tachykinin receptor 2 (*TACR2*)-expressing neurons in the medial division of the CeA were involved in fear consolidation.^36^ In another study, researchers found that protein kinase C delta (*PRKCD*) expression in the lateral division of the CeA provided inhibitory regulation in the medial division of the CeA, reducing fear expression.^37^ Similarly, through optogenetic manipulations, we demonstrated that Thy-1 cell surface antigen (*THY1*)-expressing neurons in the basolateral amygdala were involved in fear extinction and fear extinction recall.^38^,^39^

Because a generalized fear response is considered a hallmark of anxiety, researchers have examined intra-amygdala circuits and long-range projections and demonstrated that microcircuits in the amygdala play a role in anxiety. In one study, increased tonic firing of output neurons in the medial division of the CeA activated by neurons in the lateral division of the CeA was required for fear responses to the conditioned stimulus and to an unconditioned stimulus.^40^ These findings suggest that tonic activity within CeA fear circuits may be an underlying neuronal substrate for anxiety. Similarly, in the lateral amygdala, activity in distinct neuronal populations also seems to be necessary for fear generalization. One study reported that in rats that exhibited generalized fear, cells in the lateral amygdala responded to a conditioned stimulus that was not paired with an unconditioned stimulus.^41^

Because alcohol-seeking in humans has long been considered to be motivated by the desire to reduce stress and anxiety, the amygdala has been linked to behavior associated with alcohol misuse. In particular, the gamma-aminobutyric acid (GABA) neurotransmitter system in the CeA has been implicated in mediating behavior associated with acute and chronic alcohol consumption. In one study, rat brain slices exposed to an acute superfusion of ethanol increased presynaptic GABA release and enhanced postsynaptic GABA receptor function in CeA neurons.^42^ The same researchers also demonstrated that chronic ethanol exposure promoted increased basal GABA release without presynaptic effects.^43^ Furthermore, stereotactic injection of gabapentin, an anticonvulsant GABA analog, attenuated elevated operant ethanol responses in ethanol-dependent rats.^43^ Studies with transgenic mice showed that ethanol enhanced the activity of CRHR1 receptors in the CeA, implicating potential cell type–specific interactions between the stress corticotropin releasing hormone (CRH) signaling pathway and alcohol consumption and dependence.^44^ Consistent with this idea, studies have shown that rats that displayed persistent avoidance of a predator odor–paired context consumed more alcohol and exhibited compulsivelike responding for alcohol,^5^ and they expressed hyperalgesia via the CRH signaling pathway in the CeA.^45^

### PFC

The PFC, a large and complex brain region that is greatly expanded in nonhuman primates and humans, is topographically organized and has anatomically distinct subfields, roughly divided into dorsolateral, ventromedial, and orbital regions. These subfields are believed to be involved in various cognitive and emotional functions. For example, the dorsolateral regions of the PFC provide top-down regulation of attention, thought, and action and have extensive connections with sensory and motor cortices.^46^ In contrast, the ventromedial regions of the PFC regulate emotional responses and have vast connections with various subcortical structures, such as the amygdala, nucleus accumbens, and hypothalamus.^47^ The PFC also has direct and indirect interactions with the monoamine system, including noradrenergic projections from the locus coeruleus and dopaminergic inputs from the substantia nigra and VTA. The PFC is sensitive to the detrimental effects of stress exposure, as even mild uncontrolled acute stress can cause a rapid and dramatic loss of cognitive abilities, and more prolonged stress exposure causes anatomical changes in the PFC. All of these PFC pathways are critically involved in appetitive behavior, as occurs with AUD, and in emotion regulation, which is disrupted during fear processing, as occurs with PTSD.

Given the mutual connectivity between the PFC and amygdala, it has been suggested that the fortified emotional memory traces in individuals with PTSD may be a product of imbalanced interactions between the two brain areas. The PFC seems to exert an inhibitory response on the amygdala, which is a central node for emotional reactivity. In neuroimaging studies, participants with PTSD showed decreased prefrontal blood flow,^48^,^49^ and a study that used trauma reminders to provoke symptoms in patients with PTSD reported reduced activation in the ventromedial PFC.^50^ This decreased PFC activity is often accompanied by increased amygdala activity,^49^,^51^ suggesting there may be a failure of top-down cortical inhibition on the reactivation of memory traces associated with trauma-related thoughts and feelings.

The failure of top-down cortical inhibition may also relate to functional mechanisms associated with stress-related alcohol craving and relapse. Alcohol-related dysfunction in the PFC affects higher order executive function, including response inhibition and decision-making. Alcohol-related neuroadaptations in the prefrontal networks, including in the corticostriatal motivation pathways,^52^ could also promote increased relapse risk and craving for alcohol consumption. In support of these ideas, researchers have used individually calibrated, script-driven, guided-imagery procedures and neuroimaging to identify neural responses to stress and alcohol context cues.^53^,^54^ These studies demonstrated that, in healthy individuals, stress and alcohol cue exposure induced overlapping neural responses, with increased activation of the corticolimbic striatal circuit, encompassing the mPFC, orbitofrontal cortex, and anterior cingulate cortex. Healthy men displayed greater stress-induced activations throughout the prefrontal areas than healthy women, whereas women showed greater alcohol cue–related activity in the superior and middle frontal gyrus than men.^53^ These findings suggest that differential neural responses in these cortical areas may contribute to the sex differences found in stress-related coping and in vulnerabilities to stress-induced alcohol consumption and alcohol-seeking.

A follow-up study with a similar approach showed that individuals with AUD, when compared with control subjects, had less neural activity in the ventromedial PFC and anterior cingulate cortex when exposed to an alcohol-enticing or stressful stimulus.^54^ These same participants showed increased activity in the ventromedial PFC and anterior cingulate cortex during exposure to relaxing cues. These neuroimaging studies indicate that disrupted functions in the PFC, as well as in motivation-reward brain regions, may be neural mechanisms underlying alcohol craving and relapse.

Although it has been difficult to determine exactly analogous rodent and human brain regions, it is generally accepted that rodents have a PFC equivalent.^55^ Based on examination of rodent cellular structure, lamination, and projection patterns, findings suggest there are clear distinctions between the dorsal (precentral and anterior cingulate) and ventral (prelimbic, infralimbic, and medial orbital) subdivisions of the mPFC.^47^ The rodent dorsal PFC, similar to the primate PFC, is implicated in memory for motor responses, including the temporal processing of information and response selection.^56^ The ventral PFC is involved in emotional responses, such as anxiety, and in the expression and extinction of conditioned fear memory.^57^,^58^

### Hippocampus

The hippocampus is defined by its characteristic trisynaptic circuit and is well-known for its crucial roles in spatial navigation and episodic memory (i.e., recall of events within the spatial and temporal context in which they occurred).^59^ Dysfunctions of the hippocampus lead to not only memory deficits, but also anxiety, depression, epilepsy, and schizophrenia, suggesting that the hippocampus contributes to attention, arousal, and emotional states, including stress.^60^ Stress produces intense and long-lasting memories that can be a source of serious distress, but prolonged stress seems to impair performance on hippocampus-dependent memory tasks. For example, individuals diagnosed with PTSD and healthy individuals injected with cortisol (a human glucocorticoid) have been shown to be impaired in various verbal recall tests.^61^ In addition, clinical and preclinical studies have shown that stress changes synaptic plasticity and firing properties of hippocampus neurons, induces morphological atrophy, suppresses neuronal proliferation, and reduces hippocampal volume.^61^ These wide-ranging changes appear to be mediated by stress hormones. Glucocorticoids act, in part, via negative feedback of the HPA axis through the hippocampus, which is densely concentrated with glucocorticoid receptors. Similarly, rodent studies have shown that exposure to stress or high doses of corticosterone (a rodent glucocorticoid) produces deficits in hippocampus-dependent spatial memory tasks.^60^

Neuroimaging studies have demonstrated that acute alcohol exposure affects the hippocampal function of contextual or episodic memory encoding.^62^ In addition, chronic alcohol misuse seems to cause a reduction in hippocampal volume and activity.^63^,^64^ In animal studies, alcohol exposure during fetal or adolescent development has been shown to induce reductions in hippocampal neurogenesis.^65^,^66^ In addition, chronic alcohol exposure has been shown to disrupt adult hippocampal neurogenesis, alter connectivity of new neurons, and result in behavioral deficits, as demonstrated through the hippocampus-dependent novel-object recognition task and Y-maze test.^67^

### VTA and dopamine regulation

The VTA is in the midbrain, situated adjacent to the substantia nigra, and it is primarily characterized by its dopaminergic neurons, which project to limbic and cortical areas via the mesolimbic and mesocortical pathways, respectively. Electrophysiological studies in monkeys demonstrated that rewards and reward-predicting cues elicited strong phasic firing of midbrain dopamine neurons.^68^ Functional magnetic resonance imaging studies in humans have reported that increased midbrain activation occurred during anticipation of pleasant tastes^69^ and monetary gains,^70^ as well as for reward-predicting cues.^71^ Because VTA dopamine neurons project densely to the nucleus accumbens in the ventral striatum via the mesolimbic pathway, these brain areas have been implicated as major areas for processing natural rewards, reinforcement, and drugs of abuse.^72^

Studies using pharmacological perturbation and biochemical measurements have provided strong evidence for the reinforcement role of alcohol via the mesolimbic dopamine system. In a study with rats, systemic injection of dopamine receptor antagonists decreased responding for alcohol in a free-choice task, but the injection did not affect responses for water.^73^ Furthermore, in a study of nondependent rats, alcohol self-administration increased extracellular levels of dopamine in the nucleus accumbens.^74^ Such increases occurred during and also before the self-administration, indicating the motivational properties of cues associated with alcohol. Similar results have been shown in dopamine neurons of monkeys responding to reward cues.^68^

Acute exposure to different forms of stress reportedly increases dopamine release in the nucleus accumbens,^75^ whereas long-term, repeated exposure to different stressors decreases basal dopamine output in the nucleus accumbens.^76^ If the base level of dopamine has been reduced by stress, the phasic dopamine release induced by alcohol may have an amplified effect. This amplified dopamine effect may further enhance the reward-learning process, consequently leading to increases in alcohol consumption and preference.

Stress-induced alcohol preference and alcohol consumption seem to be due to alterations in both excitatory and inhibitory circuits within the VTA. A 2013 study in rats demonstrated that social isolation stress enhanced the acquisition of memories for alcohol-associated environmental cues.^77^ The learning processes were facilitated by long-term potentiation of *N*-methyl-D-aspartate (NMDA) receptor–mediated excitatory transmission in the VTA, and the facilitation could not be reversed by resocialization. In contrast, Ostroumov and colleagues showed that stress promoted alcohol use through actions on inhibitory GABA signaling in the VTA.^78^ Rats that underwent acute restraint stress 15 hours before introduction to ethanol self-administered considerably more ethanol than controls, and this increase in alcohol consumption lasted for more than 7 days. Electrophysiological recordings in the same study revealed that stress blunted the ethanol-induced increase in the firing rate of VTA dopamine neurons, which was restored by application of a GABA_A_ receptor antagonist. The stress also increased the concentration of intracellular chloride ions in VTA GABA neurons and seemed to alter the chloride gradient of GABA neurons such that, paradoxically, GABA excited these cells.

VTA dysfunction is clearly relevant to AUD. However, in PTSD, both the anhedonic component and the dopamine regulation of fear extinction may represent neuroanatomical VTA dysfunction, which may contribute to AUD and PTSD comorbidity.

## Stress Axis Function

### HPA axis

The HPA axis is the main neuroendocrine response system to stress.^61^ The activation of this system is characterized by adrenal gland synthesis and release of steroids known as glucocorticoids, such as cortisol in humans and corticosterone in rodents, triggered by the release of adrenocorticotropic hormone (ACTH) from the pituitary gland. ACTH release into the general circulation is controlled by the secretion of CRH from the paraventricular nucleus of the hypothalamus to the anterior pituitary gland via the portal blood vessels.

Glucocorticoids act on the brain through two main receptors: type I, the mineralocorticoid receptor (MR), and type II, the glucocorticoid receptor (GR). These are nuclear receptors working as transcription factors. They modulate targeted gene expression by binding to DNA or by interfering with the activity of other transcription factors.^61^ Notably, the MR has a 10-fold higher binding affinity for glucocorticoids than the GR. This differential binding affinity is assumed to create a two-tier system with negative feedback.^79^ Due to their high affinity, MRs are bounded by glucocorticoids and appear to be in a constant activated state under any physiological condition. In contrast, GRs with low binding affinity are occupied only after a significant rise of glucocorticoids. These GRs play a role in exerting negative feedback on enhanced HPA axis activity and in stress-related adaptation.^79^

As part of homeostatic processes, the actions of the HPA axis are tightly regulated to ensure that the body can optimally face stress challenges, adapt to environmental stimuli, and return to a normal state. Dysfunctions in the HPA axis frequently have been found in humans diagnosed with PTSD or AUD, so comorbidity may stem from an overlapping neurobiological mechanism. However, the details of this mechanism as a possible link between these disorders are not yet well-understood. In this section we describe recent findings on PTSD or AUD in humans and animals and how these conditions relate to the role of the HPA axis in comorbid high-stress reactivity and enhanced alcohol intake.

### Stress hormones and PTSD

Neuroendocrine studies have shown profound alterations in the HPA axis in individuals with PTSD. In particular, it has been well-documented that reduced baseline cortisol levels, in addition to enhanced cortisol suppression to a low-dose dexamethasone challenge, are present in some individuals with PTSD.^80^ These individuals also displayed augmented cortisol feedback inhibition of ACTH secretion at the level of the pituitary and a blunted ACTH response to CRH. Furthermore, because studies have consistently shown that individuals with PTSD have glucocorticoid receptor hypersensitivity, lower cortisol levels in plasma could be due to homeostatic feedback.

**Figure 1:**
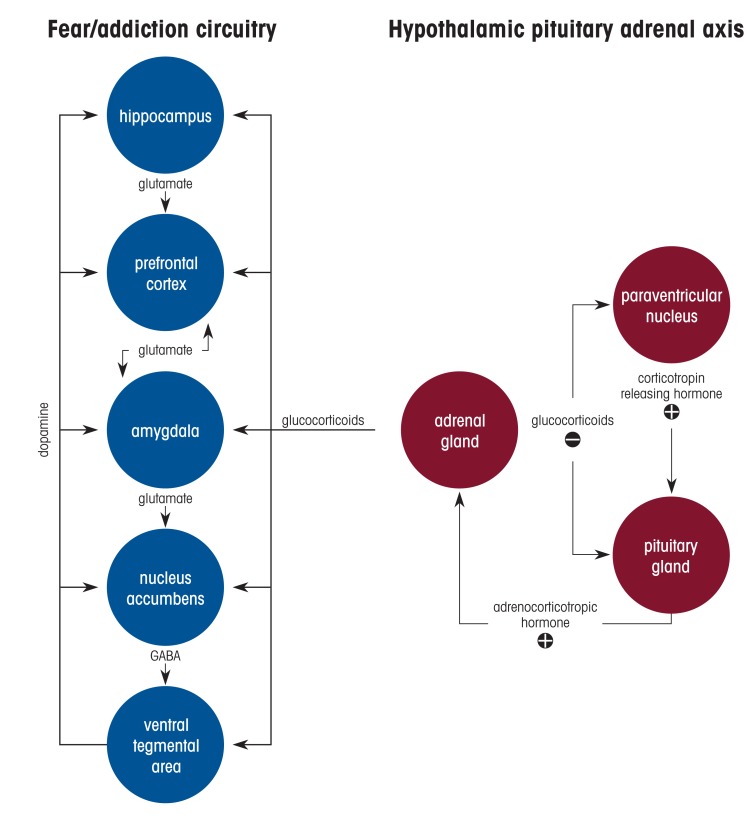
Interactions between the fear/addiction neural circuitry and the hypothalamic pituitary adrenal (HPA) axis. The fear/addiction circuitry includes the hippocampus, prefrontal cortex, amygdala, nucleus accumbens, and ventral tegmental area. The prefrontal cortex mutually connects with the amygdala, and the amygdala projects to the nucleus accumbens via its glutamatergic innervations. All these areas receive projections from dopamine neurons in the ventral tegmental area. The major components of the HPA axis include the paraventricular nucleus of the hypothalamus and the pituitary and adrenal glands. Corticotropin releasing hormone from the paraventricular nucleus stimulates adrenocorticotropic hormone (ACTH) release from the anterior pituitary into the bloodstream, then ACTH induces glucocorticoid release from the adrenal gland. Glucocorticoids mediate negative feedback in the HPA axis to reduce the stress response. Glucocorticoids also affect the fear/addiction circuitry via the glucocorticoid receptors, which triggers molecular, cellular, and physiological changes, including epigenetic alterations. *Note:* GABA, gamma-aminobutyric acid.

Glucocorticoids readily cross the blood-brain barrier, exert negative feedback at the HPA axis, and consequently reduce CRH and ACTH secretion (Figure 1). They also bind to MRs and GRs throughout the brain, including in the amygdala, hippocampus, PFC, nucleus accumbens, and septum, where they influence signaling pathways and synaptic plasticity. It has been hypothesized that different anatomical populations of GRs in the brain have unique functions in modulating plasma glucocorticoid levels. For example, in one study, application of corticosterone to the hippocampus inhibited HPA axis activation in male rats.^81^ However, in a different study, hormonal stimulation to the amygdala in rats increased plasma corticosterone and increased CRH expression in the CeA.^82^ Recent studies that used conditional knockout mouse models demonstrated that the ablation of GRs in glutamatergic, but not in GABAergic, neurons induced hyperreactivity in the HPA axis and reduced fear- and anxiety-related behavior.^83^ Furthermore, viral-mediated deletion of GRs indicated that within the basolateral amygdala glutamatergic circuits, GRs played a role in fear expression but not in anxiety. The findings suggest that fear-related behavior is modulated by GR-signaling pathways in the basolateral amygdala, whereas pathological anxiety may result from altered GR signaling in excitatory circuits in several brain areas, including the bed nucleus of the stria terminalis—which is also potentially involved in AUD and PTSD.

CRH and its receptors are expressed not only in stress-responsive areas, but also in areas of the fear- and threat-processing circuits, including in the basolateral amygdala and CeA. It has been shown that infusion of CRH or CRH binding protein into the basolateral amygdala prior to fear extinction impairs extinction recall without affecting extinction acquisition.^84^ In contrast, a CRH receptor antagonist improved extinction recall. A study that used a conditional knockout mouse model demonstrated similar results.^85^ Deletion of the alpha^1^ subunit of the GABA_A_ receptor in CRH-expressing amygdala neurons resulted in increased CRH expression in the amygdala. Consequently, anxiety behavior increased, and extinction of conditioned fear was impaired, which coincided with increased corticosterone levels in plasma.

### Stress hormones and alcohol intake

Many individuals with AUD show altered HPA axis function, raising the strong possibility that HPA axis dysfunction contributes to the development of AUD. Several studies with animal models also demonstrated that the HPA axis plays a direct role in the control of alcohol drinking. For instance, administration of corticosterone into the body or brain of rats increased their voluntary alcohol drinking, whereas administration of a corticosterone synthesis inhibitor or the removal of the adrenal glands caused decreased alcohol intake.^86^,^87^ Furthermore, a recent study demonstrated that attenuation of GR signaling reduced compulsivelike alcohol intake in alcohol-dependent rats and reduced both excessive drinking and alcohol craving in recently abstinent individuals with AUD.^88^

Given that alcohol increases dopamine release in the nucleus accumbens in animals^89^ and humans,^90^ glucocorticoids may be involved in voluntary alcohol consumption via direct action on mesocorticolimbic reward systems where GRs are abundantly expressed. A study that used a mouse model demonstrated that selective ablation of GRs in dopaminergic neurons in the brain, or of dopamine receptor D1-expressing medium spiny neurons in the striatum, highly reduced the firing rate of dopamine neurons.^91^ In the same study, mice with GR ablation in D1-expressing neurons, not in dopaminergic neurons, displayed decreased self-administration of cocaine. These findings suggest that GRs act on the postsynaptic neurons of the dopaminergic system via negative feedback from the nucleus accumbens to the VTA to increase the propensity to self-administer drugs.

In addition to the role of MRs in glucocorticoid regulation, aldosterone and MRs are the principal modulators of blood pressure and extracellular volume homeostasis via renal sodium reabsorption and potassium excretion. Although MRs are expressed in various brain areas, including in the amygdala and hippocampus, their role in stress modulation and alcohol consumption historically has received less attention. Nevertheless, recent studies with rodents, nonhuman primates, and humans have implicated the importance of the aldosterone and MR pathway in alcohol drinking and in alcohol-seeking behavior.^92^ Since MRs are also abundantly expressed in the dopaminergic system, future studies using conditional knockout mouse models are needed to determine whether these receptors contribute to alcohol intake and dependence in a manner specific to cell types or brain areas.

CRH and its receptors are also involved in alcohol behavior. In a free-choice paradigm with water and increasing concentrations of alcohol, mice lacking functional CRHR1 receptors increased alcohol intake after repeated episodes of social defeat stress.^93^ Notably, these mutant mice did not increase alcohol intake during or immediately after stress, but they did significantly increase intake 3 weeks later. Furthermore, this increased alcohol intake persisted at 6 months after the stress exposure. These findings suggest that the stress response in the HPA axis may require some time for adaptation to concurrent alcohol and stress exposure.

### Alcohol-induced stress hormone response

A large body of data suggests that alcohol is a robust activator of the HPA axis. As an example, in one study, plasma glucocorticoids in humans increased during acute and chronic alcohol consumption and during the initial phase of the alcohol withdrawal period.^94^ In another study, peripheral injection of alcohol into rats stimulated HPA axis activity, including activating the hypothalamic paraventricular nucleus, CRH release, and ACTH release.^95^

### Other neuropeptide systems associated with stress and alcohol

In addition to CRH, numerous neuropeptides have been shown in various animal models to be affected by stress or to be involved in the stress response. Studies on postmortem brain samples showed that other neuropeptides and their receptors could be suitable targets for PTSD and AUD treatments. These neuropeptides include substance P, neuropeptide Y, vasopressin, and pituitary adenylate cyclase–activating polypeptide. Progress in identifying their roles in stress and alcohol consumption has been facilitated by recent preclinical investigations, but we summarize the findings related to only two of those neuropeptides.

Substance P, with its preferred neurokinin 1 (NK1) receptor, is highly expressed in the amygdala and nucleus accumbens. Stressors induce substance P release in the amygdala, and pharmacologic blockade of NK1 receptors inhibits amygdala-associated behavioral responses in rodents.^96^ Mice genetically deficient in NK1 receptors have displayed decreased voluntary alcohol consumption and a loss of conditioned place preference for opiates.^97^,^98^ Furthermore, in a study of recently detoxified patients with AUD, treatment with an NK1 receptor antagonist suppressed spontaneous alcohol cravings and blunted cravings induced by a challenge procedure.^97^

Neuropeptide Y is well-known for opposing effects of CRH, reducing stress and anxiety, and decreasing alcohol intake in rodents. Both neuropeptides and their receptors are abundant in the amygdala and extended amygdala, including in the bed nucleus of the stria terminalis. A recent study showed that neuropeptide Y suppressed binge drinking in mice by inhibiting the activity of CRH neurons through a neuropeptide Y_1_ receptor–mediated G_i_ signaling pathway that enhances the ability of GABA to generate inhibitory currents postsynaptically.^99^ Chemogenetic activation of CRH neurons in the bed nucleus of the stria terminalis blocked the inhibitory effects of Y_1_ receptor activation on binge drinking. The same study demonstrated that chronic alcohol drinking led to persistent alterations in neuropeptide Y_1_ receptor function and suggested that shifts in the balance between neuropeptide Y and CRH might change an individual’s vulnerability to binge drinking cycles. Moreover, medications that alter this balance could be a good approach for treating binge drinking.

## Sex-Dependent Differences

Awareness is increasing regarding the crucial roles that neuronal circuits and hormones play in fear and reward processing differences between men and women. For example, researchers have reported that women suffer from anxiety and PTSD more than men,^100^ and that women use alcohol and opioids more frequently than men to handle anxiety.^53^ Although research on sex-related differences in comorbid PTSD and AUD is still in its infancy, recent clinical and preclinical studies have started disentangling the neurobiological mechanisms that may place men and women at different risk for the development of each disorder. For example, upon stress cue exposure, men display greater activation in the PFC, amygdala, and hippocampus than women, whereas women showed greater alcohol cue–related activity in brain regions associated with high-level cognitive processing.^53^ Furthermore, several studies in rodents have shown sex-related differences in neuronal morphology and in sex-hormone receptor expression in fear circuits, including in the PFC.^101^ These sex-related anatomical and molecular differences contribute to disparate functionality in the fear circuits. For example, in a rat study, researchers found that PFC function was important for fear extinction recall in males, but it was critical to fear extinction in females.^102^ Similarly, sex-related differences have been detected in the VTA dopaminergic system, and sex hormones have been implicated in differential responsiveness to drugs of abuse.^103^

## Conclusions and Future Research Needs

Epidemiological studies suggest that the diagnosis of PTSD represents a major risk factor for the development of AUD, as PTSD symptoms drive excessive alcohol consumption, and AUD worsens PTSD symptoms. Findings from the studies discussed in this article show that a vast array of neurobiological and neuroendocrine changes occur in fear/anxiety and reward/addiction circuitry, as well as in the HPA axis. Analogous changes that occur in overlapping brain areas and high rates of AUD and PTSD comorbidity suggest that these disorders share a common neurobiological etiology.

It has been extremely difficult to systematically delineate the neural basis of comorbidity. Comorbidity may be due to a conjunction of independent risk factors, shared risk factors from two disorders, or a multiform expression of one of the disorders. In this review, we focused on the comorbidity in a context in which one disorder causes the other through dysfunctions in shared neural circuitry. Since the activity of a brain area interacts with and affects other brain areas via mutually connected pathways, investigating comorbid AUD and PTSD in human and animal studies is challenging. However, the development of advanced neuroimaging has enabled an assessment of structural and functional brain network architecture at an unprecedented level of detail. New theoretical frameworks combined with network approaches are needed to focus more on the dimensional and complex nature of brain disorders.^104^

Modeling the comorbid condition in nonhuman animals is crucial, because circuit manipulations and monitoring single-neuronal activity in specific pathways and cell types will provide a better snapshot of causal relationships between PTSD and AUD. Although several studies have used rodent models to examine comorbid PTSD and AUD,^105^ preclinical studies have been challenging because of the wide array of stress procedures, different time courses of pathological behavior development, and individual differences within a model. However, technological progress in the next generation of optical, molecular, and observational tools offers a productive direction for future research using preclinical models. System-level interrogation with greater specificity may lead to identifying pathophysiological abnormalities and formulating coherent principles that explain the interactions between these disorders. Ultimately, the promise is that this knowledge may translate to hypothesis-driven, individual clinical interventions and therapeutic strategies for treating comorbid PTSD and AUD.
